# Fitness, Technical, and Kinanthropometrical Profile of Youth Lithuanian Basketball Players Aged 7–17 Years Old

**DOI:** 10.3389/fpsyg.2019.01677

**Published:** 2019-07-16

**Authors:** Kęstutis Matulaitis, Antanas Skarbalius, Catarina Abrantes, Bruno Gonçalves, Jaime Sampaio

**Affiliations:** ^1^Lithuanian Sports University, Kaunas, Lithuania; ^2^Research Center in Sport Sciences, Health and Human Development (CIDESD), University of Trás-os-Montes and Alto Douro, Vila Real, Portugal

**Keywords:** basketball, technical testing, fitness testing, long-term development, physical profile

## Abstract

Optimizing basketball performance during the stages of long-term athlete development require to identify the trainability and variation of specific technical skills, when adjusting for anthropometric changes. The aim of this study was to describe differences in height, body mass, arm span, and technical-related fitness (movement, dribbling, shooting) along the long-term development of 7–17 years Lithuanian basketball players. This cross-sectional analysis involved a total of 1051 basketball players from the Sabonis Basketball Center in Lithuania. Testing sessions were performed during 1 day of the competition period in an indoor court. The participants performed technical-related fitness tests to assess dribbling (control dribble, 20 m dribble, two balls of 20 m dribble, Illinois agility dribble), shooting (30 free-throw shoots, 1 min shooting, modified medium and long-range shots, close range shots) and defensive movements. The dribbling skills had substantial improvements (7 to 8-years-old: 20 m sprint with dribbling, effect size = 1.86; control dribble effect size = 2.18; 9 to 10-year-old: 20 m sprint with dribbling, effect size = 1.85; Illinois agility test with dribbling effect size = 1.82). Changes in defensive movement occurred mostly at the 14–15-age period. The best periods to develop dribbling and shooting skills were between 7–10 and 12–13 years, whereas defensive movements can be trained in later adolescent years. Current results and consequent normative profiles, presented as percentile tables, allow to accurately follow the players’ development.

## Introduction

The Long-term Athlete Development model outlines an appropriate training, competition and recovery program in relation to the developmental age of the individual (Balyi and Hamilton, 2004; Stafford, 2005; Balyi et al., 2009). In fact, it takes 8–12 years of training for a talented player to reach elite levels of performance and this has been elsewhere described as the 10-year or 10,000-hour rule, which translates to approximately more than 3 h of daily practice for a period of 10 years (Bloom and Sosniak, 1985; Ericsson et al., 1993; Ericsson and Charness, 1994; Salmela et al., 1998; Balyi and Hamilton, 2004). It seems clear that a specific and carefully planned training process, as well as an adequate competition and recovery regime can ensure the optimum development throughout an athlete’s career (Balyi and Hamilton, 2004). It is focused on training to optimize performance at long-term and considers sensitive developmental periods known as “windows of opportunity” (Ford et al., 2011). Developmental pathways in sport seem to be non-linear and athletes pass through discrete, but idiosyncratic stages as they develop from novices to experts (Cote and Hay, 2002; Abbott and Collins, 2004; Vaeyens et al., 2008). In basketball, although there is available research focused on the players’ pathways (Leite and Sampaio, 2010, 2012), it remains unclear when the specific skills are most sensitive to certain areas of training during their development.

Individual and collective success in basketball are well related to anthropometric and fitness characteristics (Hoare, 2000; Angyan et al., 2003). For example, anthropometric and fitness tests accounted for ∼40% in the variance of playing performance (Hoare, 2000). In fact, findings that body size and fitness are key determinants of performance in basketball are intuitive to the basketball coaching community (Drinkwater et al., 2008).

The most frequent physical and technical demands in basketball include sprints (from a few strides to over a total of 20 m), abrupt stops, fast dribbling, quick changes of movement direction, different vertical jumps, acceleration, different shots, and passes (Johnson and Nelson, 1986; Ben Abdelkrim et al., 2007; Klusemann et al., 2013). For example, differences in motor abilities of European top-quality young female basketball players were already addressed. The results showed that the body height and the technically most demanding movements performed with the ball (e.g., 20 m sprint dribble) were the most potential descriptive variables (Erculj et al., 2009, 2010). In addition, Garcia-Gil et al. (2018) used a multiple regression analysis to identify that combined age, height, contracted arm perimeter, fat skinfold thickness, and time in T-Drill test yielded a strong predictor of a performance index per time played. More recently, Ramos et al. (2019) showed the importance of maturation derived variables to achieve playing opportunities and recommended to avoid premature talent identification, providing players with opportunities to progress through the talent pathway, at least until U-16 age category. In a similar way, Guimaraes et al. (2019) showed that top players were taller, had greater fat-free mass, greater strength, power, and agility, and were technically more skillful compared with lower level players, when controlling for training experience and maturation. Also, it has been shown that performance in tests such as control dribble, speed dribble, high intensity shuttle run and dribble shuttle run, are well correlated with elite young basketball players’ power output (Apostolidis et al., 2004). In youth rugby players, a strong evaluation in performing change of direction occurs between 15 and 17 years old, because the older players seem able to perform the more advantageous “sharp” movement, instead of a “rounded” one, probably due to the positive development of basic arm and leg movements, timing and rhythmic-related abilities (Condello et al., 2013). Despite these results, research is still quite unclear about the evolution of these performance indicators across the different age groups. Therefore, the aim of this study was to identify the differences in height, body mass, arm span and technical fitness (movement, dribbling, shooting) in 7–17 years old basketball players. The outcomes may allow the establishment of normative player’s characteristic across different development stages. This information may be used as guidelines to optimize the youth players’ long-term athlete development by identifying windows of trainability and variation of specific technical skills.

## Materials and Methods

### Participants

The participants detailed profiles are presented in Table 1. They were randomly selected from the Sabonis Basketball Center youth basketball players (aged 7–17 years, *n* = 1051, between 40 and 172 in each age group).

**TABLE 1:** Structure of loads for the young Lithuanian basketball players aged 7–17 years.

**Indicators of loads**		**Age (years)**										
	
		**7**	**8**	**9**	**10**	**11**	**12**	**13**	**14**	**15**	**16**	**17**
Training per week (min)	Preparatory				4 × 90	5 × 90	5 × 90	5 × 90	5 × 90	5 × 90	5 × 90	5 × 90
	Competitive	3 × 60	3 × 90	4 × 90	5 × 90	6 × 90	6 × 90	6 × 90	6 × 90	6 × 90	7 × 90	7 × 90
	Post-Competitive				4 × 90	5 × 90	5 × 90	5 × 90	5 × 90	5 × 90	5 × 90	5 × 90
Number of training days		117	117	172	212	257	257	257	257	267	299	299
Number of training hours		117	175.5	258	318	385.5	385.5	385.5	385.5	400.5	448.5	448.5
Matches per year		0	17	36	56	63	65	65	65	66	70	72

The typical weekly workloads planned and accomplished by these young basketball players aged constantly increased for the different age groups. The number of training sessions and practice time gradually increased during each year of training (Table 2). A written informed consent was obtained from the local university institutional review board, the school principal, the subjects and their parents.

**TABLE 2:** Number of subjects in youth basketball aged 7–17 years.

**Measured Variables**	**Subjects age (years)**											**Total**
		
	**7**	**8**	**9**	**10**	**11**	**12**	**13**	**14**	**15**	**16**	**17**	
Anthropometric indicators (n)												
Height (cm)	41	46	44	90	111	83	116	138	80	61	56	857
Body mass (kg)	41	46	44	49	47	47	48	50	42	44	45	503
Arm span (cm)	41	46	44	90	96	83	116	138	80	70	56	860
Technical fitness test (n)												
Control dribble	41	42	44	73	123	105	138	135	94	55	59	909
Defensive movement	n.a.	42	44	73	93	78	105	107	71	55	59	727
20 m sprint dribble	41	45	44	46	43	n.a.	n.a.	n.a.	n.a.	n.a.	n.a.	219
Two balls of 20 m sprint dribble	n.a.	n.a.	n.a.	n.a.	n.a.	47	46	58	40	44	56	291
Illinois agility dribble	n.a.	45	44	44	43	40	46	49	41	44	45	441
30 Free-throw shooting	n.a.	n.a.	44	84	139	124	172	167	115	77	65	987
1 min shooting	n.a.	n.a.	n.a.	84	139	123	145	139	115	73	65	883
Modified medium and long-range shots	n.a.	n.a.	n.a.	n.a.	43	47	48	50	42	43	44	317
Close range shots	n.a.	42	44	49	n.a.	n.a.	n.a.	n.a.	n.a.	n.a.	n.a.	135

### Testing Procedures

The testing sessions were performed during the competitive period, and all players in the same age group were tested in an indoor court during 2 days. The players refrained from strenuous exercise for at least 48 h before the testing session. Each session was carried between 16.00 and 18.00 h by the same research team. Testing for each age group was performed in a 2 day period and during the beginning of the competitive period between October and November. In the first testing was measured the anthropometric and 20 m sprint dribble, two balls of 20 m sprint dribble and Illinois agility dribble test (around 90–110 min). The second day was dedicated to the measurement of control dribble, 30 free-throws shooting, 1 min shooting, defensive movement, close range shots, modified medium, and long range shots test of the subjects (around 90–110 min).

### Anthropometry

The subject’s body mass (to the nearest 0.1 kg, Tanita, Tanita Corporation), height without shoes (to the nearest 0.1 cm, Martin, GPM SiberHegner) and arm span using a ruler held vertically to the tape measure to record total arm span (to the nearest 0.1 cm), were measured before the participants performed a standardized warm-up for a total of 15 min. The warm up consisted of a controlled stretching routine and performing low intensity sport-specific activities using the ball, such as slow dribbling exercises.

### Technical Testing

The participants performed technical tests to assess dribbling (Control dribble, 20 m dribble, Two balls of 20 m dribble, Illinois agility dribble), shooting (30 Free-throw shooting, 1 min shooting, Modified medium and long range shots, Close range shots) and defensive movement. The participants received verbal feedback about their performance after each test and were encouraged to perform maximally in each test.

#### Control Dribble Test (Johnson and Nelson, 1986)

**FIGURE 1:**
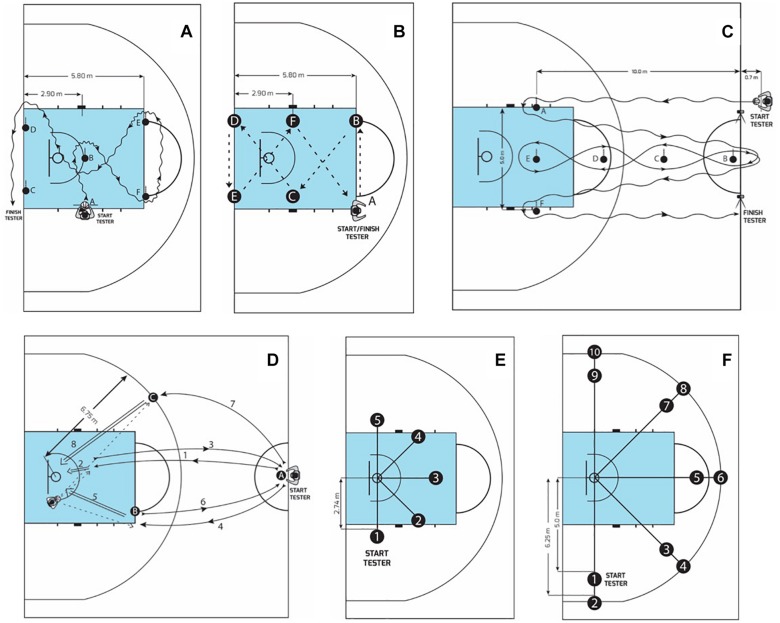
Testing protocols used: **(A)** Control dribble test; **(B)** Defensive movement test; **(C)** Illinois test with dribbling the ball; **(D)** 1 min shooting test; **(E)** Modified medium and long range shots test; **(F)** Speed spot shooting test.

Test objective: measure ball-handling skills while moving. Six cones are set up in the free-throw lane of a basketball court to provide obstacles (Figure 1A). On the signal “Ready, go,” the performer starts dribbling with the non-dominant hand from the non-dominant hand side of stand A to the non-dominant hand side of stand B (left-handed dribble). Three timed trials are given. Recovery between trials was 5 min. The best result was used for analysis.

#### Defensive Movement Test (Johnson and Nelson, 1986)

Test objective: measure basic defensive movements. The test boundaries are the free-throw line behind the basket, and the rebound lane lines. The middle-rebound lane markers serve as targets C and F for the test (Figure 1B). Additional spots outside the four corners of the rectangular area should be marked by tape (points A, B, D, and E in Figure 1B). The athlete starts at A facing away from the basket. On the signal “Ready, go,” the performer slides to the left (without crossing the feet) to marker B, touches the floor outside the lane with the left hand, performs a dropstep, and slides to point C and touches the floor outside the lane with the right hand. The athlete continues the course as shown in Figure 1B until both feet cross the finish line. Three timed trials were given. Recovery between trials was 5 min. The best performance was selected for analysis.

#### 20 m Sprint Dribbling Test

Test objective: establish and assess the speed of players while dribbling a ball. At the beginning and at the end of the 20 m distance, there were photo-electric cells connected to an electronic timer (Powertimer Testing System, NewTest, Tampere, Finland). The starting position was 70 cm from the first photo-cell. Two trials were performed with a recovery of approximately 3 min in between. The best running time was used for analysis.

#### 20 m Sprint Dribbling Two Balls

The same time recording system as in the previous 20 m sprint test was used. Each participant had one trial. If the participant lost the ball, the test was repeated up to three times. The best result was used for analysis.

#### Illinois Test With Dribbling the Ball (Getchell et al., 1998)

The same time recording system as in the 20 m sprint run was used (Figure 1C). Each participant had one trial. If the participant lost the ball, the test was repeated up to three times. The best result was used for analysis.

#### 30 Free-Throw Shooting Test (Stonkus, 2002)

Test objective: measuring the accuracy and stability skills in free-throw shooting. The subject executes a free-throw; for the first and the second shots the ball is given to a partner, after the third shot the subject takes the ball himself, dribbles it to the free-throw line and throws again. This process is repeated until 30 free-throws are taken. The test is performed once. Test result: scores the number of throws. The subject has to shoot the ball into the basket in 5 s from the moment his partner passes him the ball or he takes the ball himself and stands at the free-throw line.

#### One Minute Shooting Test (Balciunas, 2005)

Test objective: the rates of this test estimate the sensorimotor capabilities of the player, the stability of shooting along with the ability to adapt to game situations (given the quite intensive physical load and the manifestation of certain fatigue). For 1 min, the subjects were shooting from the three points distance A, B, C (close distance, middle, and long distance). On the signal “Ready, go,” the performer ran and shot from zone A, B, and C, and after each attempt the performer ran backward (Figure 1D) to the center line and the ball was passed to the shooter by another player standing under the basket. Two timed trials were given and two were recorded. Recovery between trials was 10–12 min. The best result was used for analysis.

#### Modified Medium and Long Range Shots Test (Stonkus, 1985)

Test objective: establish and measure shooting accuracy in condition of physical load. The court is marked with 10 points from which the players make shots: 1, 3, 5, 7, 9 are on the projection at 5 m distance from the center of the basketball hoop, and 2, 4, 6, 8, 10 points are at a distance of 6 m (Figure 1E). The subject stands at the first point with a ball, makes a shot, runs close to the basket, catches the scored or rebound ball, dribbles it to the second point, makes another shot, runs close to the basket and catches the ball, etc., The test includes two sets – 2 × 10 throws. The sum of throws over the limited time was registered. The duration of the test is different for young basketball players of different ages: 11–12 years – up to 145 s; 13–14 years – up to 135 s; 15–17 years – up to 130 s. For each inaccurate throw, the player gets one point if the ball falls on the hoop from above. The test was performed once.

#### Speed Spot Shooting Test (Johnson and Nelson, 1986)

Test objective: to measure skill in shooting rapidly from different positions and, to some extent, agility and ball handling. The floor markers are placed on the floor at the different spots from which the athletes must shoot. The distance of the spots from the basket is 9 foot (2.74 m). The distances for spots B, C, and D (Figure 1F) are measured from the center of the backboard: those for spots A and E are measured from the center of the basket. The athlete starts from behind any of the five markers. On the signal “Ready, go,” the person shoots, retrieves the ball and dribbles to and shoots from another spot. A maximum of four lay-up shots may be attempted, but no more than two consecutivelly. The athlete must attempt one shot from each of the five spots. Three trials of 60 s are given: the first is a practice trial, and the next two are recorded. The tester records the spots at which the shots are taken as well as the number of lay-ups attempted. Two points are given for each shot made. One point is given for any unsuccessful shot that hits the rim (from above) either initially or after bouncing from the backboard. The total points for each legal shot for each of the two trials is the score. Recovery between trials was 10–12 min. The best result was used for analysis.

A test-retest procedure was performed to assess the reliability of each test and the reliability scores are given in Table 3.

**TABLE 3:** Technical fitness test reliability.

**Test**	**Subjects age (years)**										
	
	**7**	**8**	**9**	**10**	**11**	**12**	**13**	**14**	**15**	**16**	**17**
Control dribble	0.871	0.828	0.867	0.898	0.869	0.916	0.892	0.920	0.919	0.939	0.913
Defensive movement	n.a.	0.854	0.773	0.810	0.907	0.806	0.907	0.899	0.930	0.794	0.949
20 m sprint dribble	0.857	0.732	0.857	0.963	0.954	n.a.	n.a.	n.a.	n.a.	n.a.	n.a.
Two balls of 20 m sprint dribble	n.a.	n.a.	n.a.	n.a.	n.a.	0.405	0.452	0.478	0.510	0.635	0.671
Illinois agility dribble	n.a.	0.802	0.831	0.866	0.913	0.953	0.942	0.947	0.950	0.919	0.953
30 Free-throw shooting	n.a.	n.a.	0.485	0.617	0.669	0.798	0.805	0.815	0.854	0.867	0.873
1 min shooting	n.a.	n.a.	n.a.	0.542	0.651	0.687	0.704	0.732	0.712	0.743	0.751
Modified medium and long-range shots	n.a.	n.a.	n.a.	n.a.	0.480	0.560	0.582	0.574	0.596	0.613	0.654
Close range shots	n.a.	0.765	0.572	0.653	n.a.	n.a.	n.a.	n.a.	n.a.	n.a.	n.a.

### Statistical Analysis

Descriptive data are presented graphically as means ± standard deviation. Test-retest reliability scores were obtained using intraclass correlation coefficients (ICC, two-way random effects model single measure reliability). Commercially available statistical software was used to obtain normative scores (percentile ranks) of anthropometric and technical-related fitness indicators (SPSS Inc., Version 17.0, Chicago, IL, United States). The statistical comparisons between trials were assessed using one-way repeated measures ANOVA. The magnitude from differences between age groups was assessed using standard effect sizes (Cohen, 1998; Hopkins, 2006) using previously established scales: <0.20 = trivial, 0.20–0.59 = small, 0.60–1.19 = moderate, 1.20–2.0 = large, and >2.0 = very large (Hopkins, 2002). The Pearson’s correlation coefficients were calculated to determine the relationships between the variables within each age group. Correlation coefficients with values above 0.5 were considered as representing large correlations, 0.3 to 0.5 – moderate, 0.1 to 0.3 – small and <0.1 – trivial (Cohen, 1998). The alpha level for statistical significance was set at *P* < 0.05.

## Results

### Anthropometric Indicators

**FIGURE 2:**
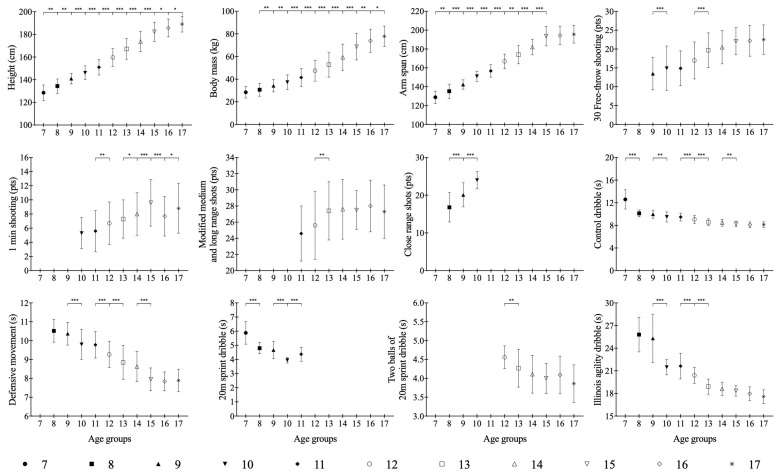
Descriptive characteristics of Lithuanian basketball players aged 7–17 years. ^*^P < 0.05; ^∗∗^*P* < 0.01; ^∗∗∗^*P* < 0.001.

Figure 2 and Table 4 present the descriptive and inferential analysis for all considered variables. Results showed that for 12 and 15-year-old basketball players, the measures that increased most were height (*ES* = 0.72–1.18, *P* < 0.001), body mass (*ES* = 0.51–0.80, *P* < 0.001) and arm span (*ES* = 0.82–1.40, *P* < 0.01). Anthropometric indicators from the subjects at the age of 16 and 17 did not change much (*ES* = 0.08–0.48).

**TABLE 4:** Inferential analysis for the characteristics of Lithuanian basketball players aged 7–17 years (absolute mean differences, percentage of variation and effect size).

**Variables**	**Subjects age (years)**									
	
	**7 vs. 8**	**8 vs. 9**	**9 vs. 10**	**10 vs. 11**	**11 vs. 12**	**12 vs. 13**	**13 vs. 14**	**14 vs. 15**	**15 vs. 16**	**16 vs. 17**
**Anthropometric indicators**										
Height (cm)	5.9*cm*	6.6*cm*	5.2*cm*	4.9*cm*	8.7*cm*	7.5*cm*	6.5*cm*	8.6*cm*	3.4*cm*	3.5*cm*
	4.38%	4.67%	3.53%	3.26%	5.42%	4.49%	3.74%	4.7%	1.83%	1.84%
	0.90(*M*)	1.20(*L*)	0.95(*M*)	0.76(*M*)	1.18(*M*)	0.89(*M*)	0.72(*M*)	0.98(*M*)	0.41(*S*)	0.46(*S*)
Body mass (kg)	2.3*kg*	3.5*kg*	3.1*kg*	4.2*kg*	6 kg	5.2*kg*	6.5*kg*	9.4*kg*	5.2*kg*	4 kg
	7.43%	10.29%	8.34%	10.14%	12.62%	9.88%	11.03%	13.65%	7.13%	5.08%
	0.42(*S*)	0.63(*M*)	0.52(*S*)	0.59(*S*)	0.70(*M*)	0.51(*S*)	0.58(*S*)	0.80(*M*)	0.48(*S*)	0.41(*S*)
Arm span (cm)	6.4*cm*	7.2*cm*	8.6*cm*	5.8*cm*	10.1*cm*	7.1*cm*	8.1*cm*	11.5*cm*	0.8*cm*	1.2*cm*
	4.73%	5.06%	5.72%	3.7%	6.02%	4.08%	4.45%	5.94%	0.4%	0.64%
	0.90(*M*)	1.16(*M*)	1.69(*L*)	0.98(*L*)	1.40(*L*)	0.82(*M*)	0.89(*M*)	1.24(*L*)	0.08(*T*)	0.13(*T*)
**Technical fitness test**										
Control dribble (s)	2.47*s*	0.15*s*	0.48*s*	0.03*s*	0.41*s*	0.5*s*	0.08*s*	0.21*s*	0.16*s*	−0.18*s*
	24.38%	1.5%	5.05%	0.32%	4.53%	5.84%	0.94%	2.54%	1.97%	−0.37%
	2.18(*VL*)	0.23(*S*)	0.60(*M*)	0.04(*T*)	0.58(*S*)	0.81(*M*)	0.14(*T*)	0.40(*S*)	0.31(*S*)	0.35(*S*)
Defensive movement (s)	*n*.*a*.	0.15*s*	0.57*s*	0.02*s*	0.51*s*	0.42*s*	0.22*s*	0.68*s*	0.11*s*	−0.05*s*
		1.45%	5.82%	0.2%	5.5%	4.75%	2.55%	8.55%	1.4%	0.63%
		0.27(*S*)	0.86(*M*)	0.03(*T*)	0.76(*M*)	0.53(*S*)	0.26(*S*)	0.98(*M*)	0.21(*S*)	0.53(*S*)
20 m sprint dribble (s)	1.08*s*	0.13*s*	0.72*s*	−0.42*s*	*n*.*a*.	*n*.*a*.	*n*.*a*.	*n*.*a*.	*n*.*a*.	*n*.*a*.
	22.5%	2.78%	18.23%	−9.61%						
	1.86(*L*)	0.28(*S*)	1.85(*L*)	1.25(*L*)						
Two balls of 20 m sprint dribble (s)	*n*.*a*.	*n*.*a*.	*n*.*a*.	*n*.*a*.	*n*.*a*.	0.29*s*	0.16*s*	0.11*s*	−0.09*s*	0.23*s*
						6.79%	3.89%	2.75%	2.2%	5.96%
						0.73(*M*)	0.35(*S*)	0.25(*S*)	0.20(*T*)	0.46(*S*)
Illinois agility dribble (s)	*n*.*a*.	0.5*s*	3.8*s*	−0.15*s*	1.27*s*	1.45*s*	0.31*s*	0.25*s*	0.38*s*	0.39*s*
		1.98%	17.83%	−0.69%	6.24%	7.67%	1.67%	1.36%	2.11%	2.22%
		0.18(*T*)	1.82(*L*)	0.11(*T*)	0.91(*M*)	1.38(*L*)	0.33(*S*)	0.31(*S*)	0.45(*S*)	0.43(*S*)
30 Free-throw shooting (pts)	*n*.*a*.	*n*.*a*.	1.4*pts*	0.0*pts*	2.1*pts*	2.7*pts*	0.8*pts*	1.6*pts*	0.1*pts*	0.3*pts*
			9.4%	0.0%	12.35%	13.71%	3.9%	7.24%	0.45%	1.33%
			0.27(*S*)	0.0(*T*)	0.44(*S*)	0.57(*S*)	0.18(*T*)	0.4(*S*)	0.03(*T*)	0.07(*T*)
1 min shooting (pts)	*n*.*a*.	*n*.*a*.	*n*.*a*.	0.3*pts*	1.1*pts*	0.6*pts*	0.7*pts*	1.6*pts*	−1.9*pts*	1.1*pts*
				5.36%	16.42%	8.22%	8.75%	16.67%	24.68%	12.5%
				0.12(*T*)	0.37(*S*)	0.21(*S*)	0.25(*S*)	0.50(*S*)	0.62(*M*)	0.35(*S*)
Modified medium and long-range shots (pts)	*n*.*a*.	*n*.*a*.	*n*.*a*.	*n*.*a*.	1 pts	1.8*pts*	0.2*pts*	−0.1*pts*	0.5*pts*	−0.7*pts*
					3.91%	6.57%	0.72%	−0.36%	1.79%	−2.56%
					0.26(*S*)	0.47(*S*)	0.05(*T*)	0.03(*T*)	0.18(*T*)	0.22(*S*)
Close range shots (pts)	*n*.*a*.	3.3*pts*	3.9*pts*	*n*.*a*.	*n*.*a*.	*n*.*a*.	*n*.*a*.	*n*.*a*.	*n*.*a*.	*n*.*a*.
		16.42%	16.25%							
		0.92(*M*)	1.41(*L*)							

The correlations between height and control dribble test were large in three age groups (8 and 16–17 years, *r* = 0.55–0.84; *P* < 0.001) and moderate in another three groups (10 and 14–15 years, *r* = 0.47–0.49; *P* < 0.001). Height was strongly correlated with the defensive movement test in 14–15 and 17 years (*r* = 0.50–0.72; *P* < 0.001) and moderately in the 9 and 16-year age groups (*r* = 0.33–0.42; *P* < 0.001).

Very large correlations were identified between arm span and the control dribble test (*r* = 0.57–0.69; *P* < 0.001) in 8, 10 and 15–17 year olds. The arm span correlated with the defensive movement test in 15–17 years (*r* = 0.52–0.68; *P* < 0.001) and with the Illinois agility test in terms of dribbling (*r* = 0.52–0.87; *P* < 0.001) in the 15 and 17 year age groups. The arm span also had large correlations with the 20 m sprint dribbling two balls test in 16 and 17 years’ age groups (*r* = 0.58–0.61; *P* < 0.001).

### Technical Fitness Determination and Assessment

In the first 4 years of training (between 7 and 10) the most notable improvement was observed in the ball dribbling skills. During the initial years of training (7–10 years old) the dribbling skills had substantial improvements (7–8 years – 20 m sprint with dribbling test *ES* = 2.176, *P* < 0.001; control dribble test *ES* = 1.862, *P* < 0.001; Illinois agility test with dribbling – 9 to 10-year-old *ES* = 1.823, *P* < 0.001). The second phase of ball dribbling skill development was at 12 and 13 years of age (*ES* = 0.91 and *ES* = 1.38, respectively, *P* < 0.001). Depending on age, the indices of shooting from close, middle and long distances changed differently. For 9 and 10 years’ (*P* < 0.001) basketball players, improvements in shooting a ball from close positions improved substantially. The greatest improvements in free throws as well as shots from medium and long range distances was between 11 and 13 years of age (Table 3). The greatest improvements in defensive movements were identified between 14 and 15 years of age (*P* < 0.001). According to the results, it can be seen that players acquired and learned dribbling skills at quickest rates, whereas shooting skills are learned later.

## Discussion

The aim of this cross-sectional analysis involving 1051 basketball players was to identify differences in height, body mass, arm span, and technical-related fitness (movement, dribbling, shooting) along the long-term development of 7–17 years basketball players.

### Body Size and Arm Span

The differences in players’ height between the ages of 7–17 years seem in line with previous findings (Norton and Olds, 2001; Ostojic et al., 2006). Cross sectional analysis of the growth spurt of Sabonis Basketball Center players showed different trends in other variables. The peak height velocity per year was identified at the age of 12 (8.66 cm) and 15 years (8.57 cm). This value is within the range of already estimated values for samples of European boys [i.e., 13.8–14.2 years (Malina et al., 2004)].

Body mass also showed a well-defined adolescent spurt, during the interval of maximum growth in weight at about 13–15 years (Malina et al., 2004). The same tendency of peak height velocity of body mass as in height was seen in basketball players at the age of 12 (6.00 kg) and 15 years (9.37 kg).

The average height and body mass of Lithuanian, American children and European boys was similar, but the body size from the subjects of current sample was higher. Correlation coefficients between height and arm span (*r* = 0.44–0.87), and between height and body mass (*r* = 0.88–0.92) were large for all players.

### Technical Fitness

In regard to the long-term basketball development, the aim of this study was to contribute to optimizing (Abbott and Collins, 2004; Bailey and Morley, 2006; Drinkwater et al., 2007) by using performance tests, indicators of change and the requirements of players at different age groups (Leonardo et al., 2002).

The technical-related fitness tests used in this study (Johnson and Nelson, 1986; Bouchard et al., 1997; Stonkus, 2002) are likely replicating the skills required in basketball games (Stonkus, 1985, 2002; Apostolidis et al., 2004). In the discussion on technical preparation it should be remembered that the manifestation of these abilities is related to the level of motor abilities (Stonkus, 1985; Karpowicz, 2006). Results for the dribble and defensive movement tests also depend on anaerobic capacity. The dribble and defensive movement indices of youth basketball players aged 7–17 years are consistent with good or very good level of technical-related fitness (Johnson and Nelson, 1986). In addition, the shooting skills showed the largest changes in the age groups of 9, 12–13, and 15 years.

The current youth basketball players have two “windows of opportunity” to improve their technical basketball skills. These periods are related to chronological age and occur at approximately 7–10 years, and 12–13 years and occur in accordance with the boys’ period of accelerated adaptation in sprint speeds, between the ages of 5 and 9 years (Borms, 1986; Viru et al., 1999). A second period of accelerated adaptation has been reported at around the age of 12 and 15 years (Borms, 1986). Additionally, the window for optimal skill training occurs between the ages of 9 and 12 years (Balyi and Hamilton, 2004; Dick, 2007).

The improvement in the technical qualifications of indicators of youth basketball players could be caused by the training program (Karpowicz, 2006; Drinkwater et al., 2007), biological maturity (Balyi and Hamilton, 2004) or genetic peculiarities (Bouchard et al., 1997). To identify and evaluate youth basketball players’ technical fitness levels at different ages it is important to establish a fitness ranking scale (Johnson and Nelson, 1986; Trninic et al., 1999; Drinkwater et al., 2008).

This study might be limited by the usage of the testing procedures also in load condition, having no possibility to account for these parameters. Further research might include the usage of session-RPE as a way to control the quality of data (see Lupo et al., 2017). Nevertheless, this study used a large sample size to identify the development in anthropometric measures and technical fitness test scores in elite Lithuanian youth players. For peak height velocity, the two most significant periods were at the ages of 12 and 15 years. The results also indicated that the best periods to develop technical skills, including dribbling and shooting, were at the ages of 7–10 years and 12–13 years, while defensive movements can be developed during 14–15 years of age. The overall results enable the establishment of normative player’s characteristic across different development stages (Supplementary Tables S1–S9), which can greatly assist coaches and researchers to design appropriate age-group strategies for training and development. This way, coaching staffs can fast and easily evaluate the players’ characteristics and their performance outputs across the developmental aging groups.

## Data Availability

All datasets generated for this study are included in the manuscript/Supplementary Files.

## Ethics Statement

The studies involving human participants were reviewed and approved by Ethics Committee from the Lithuanian Sport University. Written informed consent to participate in this study was provided by the participants’ legal guardian/next of kin.

## Author Contributions

KM, AS, and JS conceived the study. KM, AS, CA, and JS designed the methodology of the work. KM, BG, and JS analyzed the data. KM and AS drafted the manuscript. All authors reviewed and edited the manuscript.

## Conflict of Interest Statement

The authors declare that the research was conducted in the absence of any commercial or financial relationships that could be construed as a potential conflict of interest.
