# Supplementary material for: Fitness, Technical, and Kinanthropometrical Profile of Youth Lithuanian Basketball Players Aged 7–17 Years Old
Source: Front Psychol. 2019 Jul 16;10:1677. doi: 10.3389/fpsyg.2019.01677 (PMC6646714; doi:10.3389/fpsyg.2019.01677)
Supplement: Supplementary file 1 [file Data_Sheet_1.docx]

# Supplementary material

Table S1. Percentile ranks for Control dribble (s) of male youth basketball players

| Percentile | Subjects age (years) | | | | | | | | | | |
| --- | --- | --- | --- | --- | --- | --- | --- | --- | --- | --- | --- |
|  | 7 | 8 | 9 | 10 | 11 | 12 | 13 | 14 | 15 | 16 | 17 |
| 90 > | 10.78 | 9.59 | 9.01 | 8.34 | 8.58 | 8.3 | 7.81 | 7.8 | 7.65 | 7.40 | 7.80 |
| 80 | 10.89 | 9.6 | 9.23 | 8.72 | 8.72 | 8.46 | 8.04 | 7.96 | 7.85 | 7.50 | 7.90 |
| 70 | 11.65 | 9.68 | 9.49 | 9.11 | 8.98 | 8.58 | 8.19 | 8.12 | 7.97 | 7.84 | 8.08 |
| 60 | 11.73 | 9.73 | 9.73 | 9.21 | 9.15 | 8.77 | 8.30 | 8.23 | 8.10 | 7.96 | 8.10 |
| 50 | 12.25 | 9.88 | 9.85 | 9.36 | 9.39 | 8.98 | 8.50 | 8.4 | 8.19 | 8.10 | 8.20 |
| 40 | 12.41 | 10.09 | 10.11 | 9.53 | 9.50 | 9.18 | 8.67 | 8.6 | 8.25 | 8.26 | 8.30 |
| 30 | 12.76 | 10.2 | 10.35 | 9.72 | 9.80 | 9.43 | 8.80 | 8.8 | 8.50 | 8.35 | 8.40 |
| 20 | 13.38 | 10.45 | 10.69 | 10.24 | 10.19 | 9.56 | 9.10 | 8.9 | 8.70 | 8.50 | 8.58 |
| < 10 | 15.46 | 10.86 | 10.82 | 10.52 | 10.50 | 9.9 | 9.38 | 9.11 | 8.90 | 8.74 | 8.84 |
| Range | 10.58–16.34 | 9.48–11.88 | 8.69–11.72 | 7.98–12.05 | 8.10–11.70 | 7.82–11.10 | 7.37–9.90 | 7.13–10.40 | 6.92–9.50 | 7.11–9.40 | 7.50–9.59 |

Table S2. Percentile ranks for Defensive movement (s) of male youth basketball players

| Percentile | Subjects age (years) | | | | | | | | | |
| --- | --- | --- | --- | --- | --- | --- | --- | --- | --- | --- |
|  | 8 | 9 | 10 | 11 | 12 | 13 | 14 | 15 | 16 | 17 |
| 90 > | 9.67 | 9.59 | 8.88 | 9 | 8.3 | 7.81 | 7.72 | 7.27 | 7.3 | 7.4 |
| 80 | 9.69 | 9.66 | 9.04 | 9.2 | 8.7 | 8.04 | 8.09 | 7.7 | 7.42 | 7.7 |
| 70 | 10.3 | 9.94 | 9.32 | 9.4 | 8.9 | 8.25 | 8.2 | 7.54 | 7.53 | 7.8 |
| 60 | 10.32 | 10.15 | 9.63 | 9.6 | 9.03 | 8.4 | 8.28 | 7.73 | 7.7 | 8 |
| 50 | 10.43 | 10.36 | 9.84 | 9.7 | 9.26 | 8.66 | 8.4 | 7.9 | 7.83 | 8.11 |
| 40 | 10.5 | 10.57 | 9.94 | 9.8 | 9.4 | 8.97 | 8.67 | 8.09 | 7.9 | 8.2 |
| 30 | 10.79 | 10.69 | 10.05 | 10.03 | 9.5 | 9.3 | 8.9 | 8.16 | 8.01 | 8.3 |
| 20 | 11.08 | 10.78 | 10.39 | 10.3 | 9.79 | 9.6 | 9.17 | 8.4 | 8.2 | 8.61 |
| < 10 | 11.36 | 11.09 | 10.92 | 10.6 | 10.3 | 10.2 | 9.6 | 8.78 | 8.49 | 8.7 |
| Range | 9.61–11.46 | 9.43–11.59 | 8.13–11.55 | 8.29–11.9 | 7.8–11.2 | 7.29–11.1 | 7.1–11.6 | 6.6–9.53 | 6.5–8.89 | 6.7–10.2 |

Table S3. Percentile ranks for *20m sprint dribble* (s) of male youth basketball players

| Percentile | Subjects age (years) | | | | |
| --- | --- | --- | --- | --- | --- |
|  | 7 | 8 | 9 | 10 | 11 |
| 90 > | 4.95 | 4.25 | 4.04 | 3.66 | 3.72 |
| 80 | 5.03 | 4.55 | 4.12 | 3.78 | 3.92 |
| 70 | 5.24 | 4.58 | 4.3 | 3.82 | 4.17 |
| 60 | 5.39 | 4.64 | 4.42 | 3.87 | 4.24 |
| 50 | 5.81 | 4.69 | 4.5 | 3.91 | 4.36 |
| 40 | 6.09 | 4.76 | 4.71 | 3.99 | 4.49 |
| 30 | 6.14 | 4.95 | 4.83 | 4.04 | 4.59 |
| 20 | 6.35 | 5.13 | 4.9 | 4.08 | 4.76 |
| < 10 | 6.89 | 5.3 | 5.65 | 4.17 | 4.85 |
| Range | 4.89–7.52 | 4.23–6.02 | 3.93–6.18 | 3.49–4.68 | 3.42–5.47 |

Table S4. Percentile Ranks for *Two balls of 20m sprint dribble (s)* of male youth basketball players

| Percentile | Subjects age (years) | | | | | |
| --- | --- | --- | --- | --- | --- | --- |
|  | 12 | 13 | 14 | 15 | 16 | 17 |
| 90 > | 4.18 | 3.63 | 3.53 | 3.44 | 3.56 | 3.4 |
| 80 | 4.22 | 3.79 | 3.71 | 3.66 | 3.66 | 3.45 |
| 70 | 4.26 | 3.92 | 3.77 | 3.71 | 3.74 | 3.54 |
| 60 | 4.43 | 4.04 | 3.87 | 3.85 | 3.81 | 3.55 |
| 50 | 4.46 | 4.23 | 4 | 3.95 | 4 | 3.61 |
| 40 | 4.52 | 4.39 | 4.15 | 3.99 | 4.06 | 3.85 |
| 30 | 4.73 | 4.48 | 4.41 | 4.04 | 4.25 | 3.97 |
| 20 | 4.85 | 4.72 | 4.59 | 4.28 | 4.31 | 4.17 |
| < 10 | 5.08 | 4.88 | 4.74 | 4.45 | 4.82 | 4.47 |
| Range | 4.13–5.42 | 3.44–5.06 | 3.44–5.27 | 3.39–5.08 | 3.45–5.46 | 3.38–5.34 |

Table S5. Percentile ranks for Illinois agility dribble (s) of male youth basketball players

| Percentile | Subjects age (years) | | | | | | | | | |
| --- | --- | --- | --- | --- | --- | --- | --- | --- | --- | --- |
|  | 8 | 9 | 10 | 11 | 12 | 13 | 14 | 15 | 16 | 17 |
| 90 > | 23 | 21.45 | 16.69 | 19.59 | 18.6 | 17.4 | 17.34 | 17.36 | 16.88 | 16.75 |
| 80 | 23.81 | 21.85 | 20.88 | 19.88 | 19.11 | 17.91 | 17.57 | 17.43 | 17.31 | 16.96 |
| 70 | 24.16 | 22.99 | 21.05 | 20.72 | 19.64 | 18.15 | 18.06 | 17.76 | 17.52 | 16.98 |
| 60 | 24.44 | 24.26 | 21.2 | 21.04 | 20.17 | 18.31 | 18.37 | 18.16 | 17.56 | 17.1 |
| 50 | 24.84 | 24.97 | 21.41 | 21.3 | 20.41 | 19.06 | 18.49 | 18.21 | 17.73 | 17.37 |
| 40 | 25.9 | 25.78 | 21.75 | 21.38 | 20.59 | 19.23 | 18.71 | 18.52 | 17.78 | 17.6 |
| 30 | 27.19 | 26.83 | 21.89 | 22.15 | 20.9 | 19.31 | 18.96 | 18.73 | 17.79 | 17.87 |
| 20 | 27.62 | 27.08 | 22.28 | 22.78 | 21.27 | 19.71 | 19.46 | 18.76 | 18.27 | 18.07 |
| < 10 | 28.12 | 29.2 | 22.6 | 24.07 | 21.56 | 20.35 | 19.67 | 19.25 | 19.02 | 18.3 |
| Range | 22.8–32.65 | 20.72–35.9 | 19.2–23.57 | 19.13–26.38 | 18.68–22.5 | 16.81–20.93 | 17.14–20.83 | 17.31–20.1 | 16.79–21.08 | 16.68–20.1 |

Table S6. Percentile ranks for 30 Free-throw shooting (pts) of male youth basketball players

| Percentile | Subjects age (years) | | | | | | | | |
| --- | --- | --- | --- | --- | --- | --- | --- | --- | --- |
|  | 9 | 10 | 11 | 12 | 13 | 14 | 15 | 16 | 17 |
| 90 > | 20 | 23 | 21 | 24 | 25 | 26 | 27 | 28 | 28 |
| 80 | 19 | 22 | 19 | 22 | 24 | 25 | 26 | 27 | 27 |
| 70 | 16 | 20 | 18 | 20 | 23 | 24 | 25 | 26 | 25 |
| 60 | 15 | 16 | 17 | 19 | 22 | 23 | 24 | 24 | 24 |
| 50 | 14 | 15 | 16 | 17 | 21 | 22 | 23 | 23 | 23 |
| 40 | 13 | 13 | 14 | 16 | 20 | 20 | 22 | 22 | 22 |
| 30 | 11 | 12 | 13 | 15 | 19 | 19 | 21 | 20 | 21 |
| 20 | 10 | 10 | 11 | 13 | 17 | 18 | 20 | 19 | 20 |
| < 10 | 8 | 7 | 9 | 11 | 13 | 14 | 17 | 17 | 17 |
| Range | 5–21 | 3–28 | 4–24 | 5–27 | 4–28 | 5–29 | 11–29 | 13–29 | 13–30 |

Table S7. Percentile ranks for 1 min shooting (pts) of male youth basketball players

| Percentile | Subjects age (years) | | | | | | | |
| --- | --- | --- | --- | --- | --- | --- | --- | --- |
|  | 10 | 11 | 12 | 13 | 14 | 15 | 16 | 17 |
| 90 > | 10 | 10 | 12 | 11 | 13 | 14 | 12 | 14 |
| 80 | 9 | 9 | 10 | 10 | 11 | 13 | 11 | 13 |
| 70 | 8 | 8 | 9 | 9 | 10 | 12 | 10 | 11 |
| 60 | 7 | 7 | 8 | 8 | 9 | 11 | 9 | 10 |
| 50 | 6 | 6 | 7 | 7 | 8 | 10 | 8 | 9 |
| 40 | 5 | 5 | 6 | 6 | 7 | 9 | 7 | 8 |
| 30 | 4 | 4 | 5 | 5 | 6 | 8 | 6 | 7 |
| 20 | 3 | 3 | 4 | 4 | 5 | 7 | 5 | 6 |
| < 10 | 2 | 2 | 3 | 3 | 4 | 6 | 4 | 5 |
| Range | 1–13 | 1–16 | 0–16 | 2–15 | 2–16 | 2–18 | 2–13 | 2–17 |

Table S8. Percentile ranks *Modified medium and long range shots* (pts) of male youth basketball players

| Percentile | Subjects age (years) | | | | | | |
| --- | --- | --- | --- | --- | --- | --- | --- |
|  | 11 | 12 | 13 | 14 | 15 | 16 | 17 |
| 90 > | 30 | 32 | 32 | 34 | 33 | 34 | 32 |
| 80 | 28 | 30 | 31 | 31 | 30 | 32 | 31 |
| 70 | 27 | 29 | 30 | 30 | 29 | 31 | 30 |
| 60 | 26 | 28 | 29 | 29 | 28 | 29 | 29 |
| 50 | 25 | 26 | 28 | 28 | 27 | 28 | 28 |
| 40 | 24 | 25 | 27 | 27 | 26 | 27 | 27 |
| 30 | 23 | 24 | 26 | 26 | 25 | 26 | 26 |
| 20 | 22 | 23 | 25 | 25 | 24 | 25 | 25 |
| < 10 | 21 | 21 | 23 | 24 | 23 | 24 | 23 |
| Range | 18–33 | 14–35 | 19–34 | 17–36 | 22–34 | 22–35 | 18–33 |

Table S9. Percentile ranks Close range shots *(pts)* of male youth basketball players

| Percentile | Subjects age (years) | | |
| --- | --- | --- | --- |
|  | 8 | 9 | 10 |
| 90 > | 22 | 25 | 28 |
| 80 | 19 | 24 | 27 |
| 70 | 18 | 23 | 26 |
| 60 | 17 | 22 | 25 |
| 50 | 16 | 21 | 24 |
| 40 | 15 | 20 | 23 |
| 30 | 14 | 19 | 22 |
| 20 | 13 | 18 | 21 |
| < 10 | 11 | 16 | 20 |
| Range | 10–25 | 14–26 | 19–29 |
